# Effects of a biologic agent in a patient with rheumatoid arthritis after treatment for methotrexate-associated B-cell lymphoma: a case report

**DOI:** 10.1186/1756-0500-7-229

**Published:** 2014-04-11

**Authors:** Takeshi Kuroda, Hiroe Sato, Takeshi Nakatsue, Yoko Wada, Shuichi Murakami, Masaaki Nakano, Ichiei Narita

**Affiliations:** 1 Niigata University Health Administration Center, 2-8050 Ikarashi, Nishi-ku, Niigata City 950-2181, Japan; 2 Division of Clinical Nephrology and Rheumatology, Niigata University Graduate School of Medical and Dental Sciences, 1-757 Asahimachi-Dori, Chuo-ku, Niigata City 951-8510, Japan; 3 Department of Medical Technology, School of Health Sciences, Faculty of Medicine, Niigata University, 2-746 Asahimachi-Dori, Chuo-ku, Niigata City 951-8518, Japan

**Keywords:** Rheumatoid arthritis, Lymphoma, Biologics

## Abstract

**Background:**

Several studies have suggested an increased risk of malignant tumor in patients with rheumatoid arthritis. It has been also reported that rheumatoid arthritis patients have a high incidence of lymphoma compared with the general population, and that patients receiving methotrexate, which is the anchor drug for rheumatoid arthritis treatment, can develop lymphoproliferative disease. Nevertheless, management of rheumatoid arthritis after treatment for methotrexate-associated lymphoma has not been fully investigated. We here report a patient with rheumatoid arthritis who developed malignant lymphoma associated with methotrexate therapy. Moreover, we describe the use of a biologic agent for a rheumatoid arthritis patient after treatment for lymphoma associated with methotrexate.

**Case presentation:**

A 60-year-old Japanese man with a 20-year history of rheumatoid arthritis was admitted to our hospital with a left inguinal tumor. Open biopsy was performed and a biopsy specimen revealed diffuse large B-cell lymphoma. As our patient had received methotrexate for 4 years, we diagnosed the lymphoproliferative disease as being methotrexate-related. This lymphoma was not associated with Epstein- Barr virus by Epstein-Barr virus-encoded ribonucleic acid in-situ hybridization, but this patient was an Epstein-Barr virus carrier, regarding serological testing. The lymphoma went into complete remission after 6 courses of rituximab plus cyclophosphamide, hydroxydaunorubicin, vincristine, and prednisone/prednisolone therapy. Two years later, however, rheumatoid arthritis activity gradually increased and was not controlled with salazosulfapyridine. Etanercept was administered in view of its possible effect on B-cells, and this reduced the level of disease activity without recurrence of lymphoma.

**Conclusion:**

The management of rheumatoid arthritis after treatment for methotrexate-associated lymphoma has not been fully investigated yet. Etanercept appeared to be safe because of its B-cell effect, but further observation is necessary to make a firm conclusion. Further accumulation of cases is needed to clarify which biologics are safe and effective for treatment of methotrexate-associated B-cell lymphoma.

## Background

Rheumatoid arthritis (RA) is a chronic inflammatory disease characterized by painful swollen joints, impaired mobility of the affected joints and permanent damage to the cartilage and bone. Methotrexate (MTX) is an anchor drug in the treatment of RA, and has been shown to delay the progression of radiographic changes in the joints, halt worsening of the quality of life, and prolong the life span of patients with RA [1,2].

However, a proportion of patients receiving MTX therapy may develop potentially life-threatening adverse events such as interstitial pneumonia [3-9], severe bone marrow suppression [10,11] and lymphoproliferative disease (LPD), including malignant lymphoma [12-15]. Until now, there has been some debate over whether MTX therapy for RA patients is associated with an increasing risk of developing lymphoma [16-18].

Here we report a patient with RA who sequentially developed diffuse large B-cell lymphoma (DLBCL) during a 4-year course of MTX therapy. We also discuss the clinical effects and safety of biologics after treatment of lymphoma.

## Case presentation

A 60-year-old Japanese man with a 20-year history of RA was admitted to our hospital with a left inguinal tumor in May 2011. His family history included no consanguinity or collagen diseases. He had first developed polyarthralgia in March 2003, and visited our satellite hospital. A diagnosis of RA was made, based on the presence of symmetrical polyarthritis involving the hands, elbows and knees, and positivity for serum rheumatoid factor (RF). Initially he was treated with bucillamine (100 mg/day) and prednisolone (2.5 mg/day), but this was soon switched to salazosulfapyridine (500 mg/day). His RA disease activity temporarily subsided, but later flared up again in May 2007. In June 2007, MTX was substituted for salazosulfapyridine at the dose of 6 mg/week. Treatment with tacrolimus was added in December 2008 at a dose of 1 mg daily, and was soon increased to 2 mg daily. Tacrolimus was switched to mizoribine (100 mg/day) in March 2009, because the arthritis was not controlled. Therefore, the dose of MTX (8 mg/week) was increased along with mizoribine (8 mg/week) in November 2011. The patient showed gradual resolution of his articular symptoms in response to MTX.

**Figure 1:**
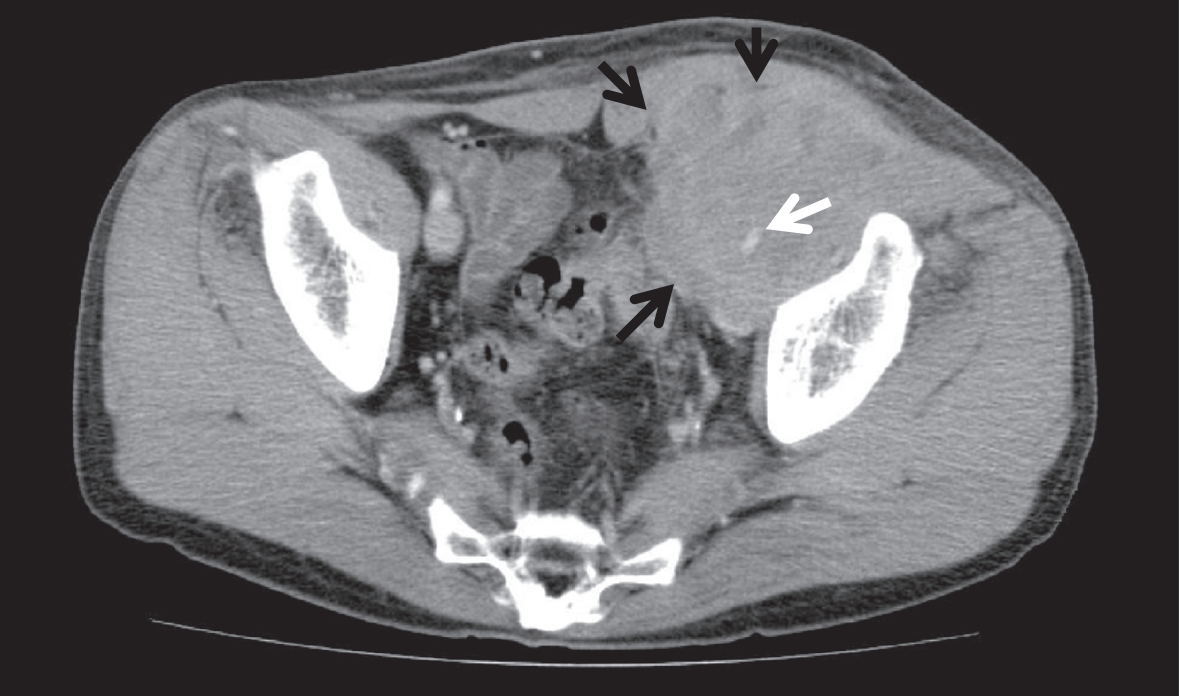
Abdominal contrast computed tomography revealed that a mass, approximately 7.0 cm in diameter (black arrows), was detected in the left inguinal region and the tumor involved the external iliac vein (white arrow).

In April 2011, he noticed a mass about 3 cm in diameter in his left inguinal region, and this increased rapidly in size over the next month. Abdominal contrast computed tomography (CT) revealed a mass, approximately 7.0 cm in diameter, in the left inguinal region and involving the external iliac vein (Figure 1). Additionally, there was a thrombus in the distal part of the left external iliac vein: therefore, he was referred to our hospital on May 31. While scheduled to undergo a biopsy of the mass, he was admitted on May 31. At that time, there was no evidence of active synovitis.

On physical examination, his blood pressure was 120/62 mmHg with a regular heartbeat of 80 bpm and a temperature of 36.0°C. Pulse oximetry showed an oxygen saturation of 98%. Cardiac, lung and abdominal examination revealed no abnormalities. Marked left foot edema was observed. Neurological examination revealed no abnormalities. There was symmetric polyarthritis in the proximal interphalangeal and metacarpophalangeal joints of the hand, wrist, and ankle. Laboratory studies revealed a leukocyte count of 5740 per mm^3^, a red blood cell count of 387 × 10^4^ per mm^3^, a hematocrit of 39.2%, hemoglobin 12.7 g/dL, platelet count 22.9 × 10^4^ per mm^3^, and a C-reactive protein level of 11.35 mg/dL. Electrolytes were normal with a total protein level of 7.1 g/dL and hypoalbuminemia (2.9 g/dL). The blood urea nitrogen concentration was 13.0 mg/dL, and the serum creatinine level was 0.68 mg/dL. Urinalysis showed a urinary protein excretion of 0.1 g/day, and no hematuria. His level of RF was 127.0 IU/mL and anti-cyclic citrullinated peptide antibody was 801.8 U/mL; antinuclear antibodies were negative. Blood tests revealed the following values: alanine transaminase 90 IU/L, aspartate aminotransferase 71 IU/L, alkaline phosphatase 608 IU/L, lactate dehydrogenase 394 IU/L, glutamyl transaminase 187 IU/L, total bilirubin 2.8 mg/dL, total cholesterol 196 mg/dL, and triglyceride 113 mg/dL. The serum immunoglobulin (Ig) levels were as follows: IgG: 1171 mg/dL; IgA: 401 mg/dL; IgM: 65 mg/dL. The serum carcinoembryonic antigen level was 1.5 ng/mL (normal range, 1.0–6.7 ng/mL), squamous cell carcinoma antigen level 0.5 ng/mL (normal range, <1.5 ng/mL), carbohydrate antigen-125 24.0 ng/mL (normal range, <35 ng/mL), α-fetoprotein 3.0 ng/mL (normal range <10 ng/mL), prostate specific antigen 0.54 ng/mL (normal range <3.0 ng/mL), and carbohydrate antigen 19-9 5 U/mL (normal range, <37 U/mL). The soluble interleukin-2 receptor level was elevated to 1168 U/mL (normal range, 220–530 U/mL). Notably, serum IgG reaction with Epstein-Barr virus (EBV)-viral capsid antigen (VCA) was positive (1/160), serum IgA reaction with EBV VCA was negative (<1/10), serum IgM reaction with EBV VCA was negative (<1/10), serum IgG reaction with EBV-early antigen (EBV-EA) was negative (<1/10), serum IgA reaction with EBV-EA was negative (<1/10), and that with the nuclear antigen was positive (1/20) (normal range, <1/10). Additionally, chest X-ray showed no abnormalities.

A CT scan of the abdomen and chest showed no ascites and no lymphadenopathy except for the left inguinal tumor with involvement of the left external iliac artery. Histological analysis of a biopsied lymph node revealed DLBCL with cluster of differentiation (CD) 20^+^, CD3^-^ and negativity for EBV-encoded ribonucleic acid (EBER) in-situ hybridization (ISH). Flow cytometry analysis showed a pathological cell phenotype of CD19^+^, CD20^+^, CD22^+^, CD5^-^, CD10^-^ and B-cell type. Cytogenetic analysis detected 45, X, -Y [3] /46, XY [17], indicating deletion of chromosome Y. After diagnosis of lymphoma, the patient received six courses of chemotherapy (R-CHOP: rituximab, cyclophosphamide, adriamycin, vincristine, prednisone). After the first chemotherapy cycle, his foot edema began to improve and the left inguinal tumor suddenly became non-palpable. CT scans of the abdomen and chest taken in July 2011 revealed complete resolution of the lymph node enlargement, and complete remission of the DLBCL. He was discharged from hospital in July 2011, and followed up as an outpatient. His RA activity then gradually increased with symmetrical polyarthritis involving the hands, elbows and knees, and therefore salazosulfapyridine (1000 mg/day) and prednisolone (10 mg/day) were readministered. The RA disease activity became temporarily quiescent, but later flared up again in March 2013. Therefore, in May 2013, etanercept was added at 25 mg twice a week without MTX to control the arthritis, and thereafter the RA activity gradually improved. The disease activity score 28- erythrocyte sedimentation rate (DAS28-ESR) was 2.8 in August 2013 without any side effects or recurrence of the lymphoma.

## Conclusions

Several studies have suggested an increased risk of malignant tumor in patients with RA [19-21], but the majority of reports have noted that the risk is not increased to a significant degree [22-26]. Whether or not RA patients treated with MTX have an increased risk of developing lymphoma remains a matter of controversy. It has been reported that RA patients have a high incidence of lymphoma compared with the general population, and that patients receiving MTX, which is the anchor drug for RA treatment, can develop LPD [12]. The risk of malignant lymphoma in patients with RA has been estimated to be 1.9–6.7 times that in the general population [20,21,24,26,27]. Recently, several reports have suggested that EBV infection might be related to LPD associated with MTX [13,28]. EBV is a B-lymphocytotropic virus causing a variety of B-cell disorders, including infectious mononucleosis and LPD in patients who are immunosuppressed. Moreover, other reports have indicated that many spontaneous malignant lymphomas have become attenuated by withdrawal of MTX treatment alone [29,30]. These reports clearly indicate that MTX induces LPD. However, the relationship between the duration of MTX treatment and the onset of LPD [18,31] still remains unclear. Several epidemiological studies have failed to find any overall association between MTX exposure and the development of lymphoma in RA patients [18,32-34]. By contrast, it has been reported that the risk of lymphoma is higher in RA patients with high disease activity [35]. Thus, it is considered that MTX treatment reduces the disease activity of RA, leading to suppression for LPD, whereas MTX treatment induces LPD and EBV-related LPD in immunosuppressed patients. With regard to the histopathological type of lymphoma, a previous report has revealed that 78% of RA patients have B-cell lymphoma, 5% have T–natural killer-cell lymphoma, and 6% have Hodgkin’s disease; 48% of B-cell malignant lymphomas in RA patients were been identified as DLBCL, which is the most common form [35]. In our patient the lymphoma was diagnosed as DLBCL and the disease activity of RA could not be sufficiently controlled. As our patient had received MTX for 4 years, we diagnosed the LPD as being MTX-related. This lymphoma was not associated with EBV by EBER-ISH, but this patient was an EBV carrier, regarding serological test.

As for anti-tumor necrosis factor (TNF) therapy, the association between anti-TNF therapy and malignant lymphoma was also unclear. Several reports have indicated that patients receiving anti-TNF antibody therapy had no significantly increased risk of developing malignant lymphoma [21,36]. However, another report has indicated that patients receiving anti-TNF antibody therapy had a significantly higher incidence of lymphoma than patients without anti-TNF antibody therapy [37]. According to a meta-analysis of nine large-scale clinical trials involving RA patients, the risk of developing a malignant tumor was 3.3 times higher than in controls [38]. There was considerable variation among individual cases, but the situation was complicated by several factors, and epidemiologic trends were unclear. After treatment for malignant lymphoma, our patient achieved complete remission with rituximab, but the disease activity of RA gradually became higher again. In this case, we determined that MTX was contraindicated and administered salazosulfapyridine first, but it was not effective. Therefore we decided to use biologics.

The rationale for treatment of lymphoma with a TNF inhibitor is still unclear. Although a direct action of TNF or anti-TNF on B-cells has been hypothesized, no increase in survival or apoptosis has been found [18,39]. Anti-TNF agents can be structurally classified into two types; one type is generated as an antibody against human TNF-α and the other type is engineered from human TNF receptors. The former type includes infliximab, adalimumab, and golimumab, and the latter type is etanercept. Biologic treatments are known to affect B-cells. Anti-TNF antibodies such as infliximab and adalimumab show complement-dependent cytotoxicity (CDC), antibody-dependent cell-mediated cytotoxicity (ADCC), and outside-to-inside signaling (apoptosis/cell cycle arrest) via transmembrane TNF-α, whereas etanercept has only ADCC activity and lacks CDC activity and outside-to-inside signaling via transmembrane TNF-α in vitro [40]. Transmembrane TNF-α is also known to exert unique biologic functions such as cytotoxic activity and polyclonal B-cell activation, in a cell-to-cell contact manner. Tocilizumab, an anti-interleukin-6 receptor inhibitor, reduces the frequency of peripheral preswitching and post-switch memory B-cells [41]. For this reason, we did not choose tocilizumab for the present patient. Additionally, it has been reported that both abatacept [42], a fusion protein composed of immunoglobulin fused to the extracellular domain of cytotoxic T-lymphocyte antigen 4, and certolizumab pegol [43], a humanised anti-TNF pegylated Fab’ fragment do not mediate CDC and ADCC. Considering the features of these biologics and the fact that our patient had DLBCL with B-cell surface markers, we thought that etanercept would be safer than other biologics. Our patient achieved low disease activity without recurrence of malignant lymphoma after 5 months of treatment. If the response with etanercept is lost, abatacept or certolizumab pegol might be chosen.

Here we have described the use of a biologic agent for an RA patient after treatment for B-cell lymphoma associated with MTX. We chose etanercept for our patient considering its influence on B-cells, and he achieved low disease activity. Etanercept appeared to be safe because of its B-cell effect, but further observation is necessary to make a firm conclusion. Further accumulation of cases is needed to clarify which biologics are safe and effective in this situation.

## Consent

Written informed consent was obtained from the patient for publication of this case report and any accompanying images. A copy of the written consent is available for review from the Editor-in-Chief of this journal.

## Competing interests

The authors declare that they have no competing interests.

## Authors’ contributions

TK, HS, TN, YW and SM made substantial contributions to the conception and design, and the acquisition, analysis, and interpretation of data. They were also involved in drafting and revising the manuscript. MN and IN contributed to the conception and design, and performed critical revision of the manuscript. All authors read and approved the final manuscript.
